# Minireview Exploring the Biological Cycle of Vitamin B3 and Its Influence on Oxidative Stress: Further Molecular and Clinical Aspects

**DOI:** 10.3390/molecules25153323

**Published:** 2020-07-22

**Authors:** Bogdan Doroftei, Ovidiu-Dumitru Ilie, Roxana-Oana Cojocariu, Alin Ciobica, Radu Maftei, Delia Grab, Emil Anton, Jack McKenna, Nitasha Dhunna, Gabriela Simionescu

**Affiliations:** 1 Faculty of Medicine, University of Medicine and Pharmacy “Grigore T. Popa”, University Street, no 16, 700115 Iasi, Romania; bogdan.doroftei@umfiasi.ro (B.D.); delianicolaiciuc@yahoo.com (D.G.); emil.anton@yahoo.com (E.A.); gabi.ginecologie@gmail.com (G.S.); 2 Clinical Hospital of Obstetrics and Gynecology “Cuza Voda”, Cuza Voda Street, no 34, 700038 Iasi, Romania; 3 Origyn Fertility Center, Palace Street, no 3C, 700032 Iasi, Romania; 4 Department of Research, Faculty of Biology, Alexandru Ioan Cuza University, Carol I Avenue, no 11, 700505 Iasi, Romania; ovidiuilie90@yahoo.com (O.-D.I.); roxana_20_2006@yahoo.com (R.-O.C.); 5 York Hospital, Wigginton road Clifton, York YO31 8HE, UK; jackmckenna@doctors.org.uk; 6 Mid Yorkshrie Hospitals NHS Trust, Pinderfields Hospital, Wakefield WF1 4DG, UK; nitasha.dhunna@doctors.org.uk

**Keywords:** niacin, nicotinamide adenine dinucleotide, DNA, oxidative stress, gastrointestinal microflora, oocyte quality, SARS-CoV-2

## Abstract

Vitamin B3, or niacin, is one of the most important compounds of the B-vitamin complex. Recent reports have demonstrated the involvement of vitamin B3 in a number of pivotal functions which ensure that homeostasis is maintained. In addition, the intriguing nature of its synthesis and the underlying mechanism of action of vitamin B3 have encouraged further studies aimed at deepening our understanding of the close link between the exogenous supply of B3 and how it activates dependent enzymes. This crucial role can be attributed to the gut microflora and its ability to shape human behavior and development by mediating the bioavailability of metabolites. Recent studies have indicated a possible interconnection between the novel coronavirus and commensal bacteria. As such, we have attempted to explain how the gastrointestinal deficiencies displayed by SARS-CoV-2-infected patients arise. It seems that the stimulation of a proinflammatory cascade and the production of large amounts of reactive oxygen species culminates in the subsequent loss of host eubiosis. Studies of the relationhip between ROS, SARS-CoV-2, and gut flora are sparse in the current literature. As an integrated component, oxidative stress (OS) has been found to negatively influence host eubiosis, in vitro fertilization outcomes, and oocyte quality, but to act as a sentinel against infections. In conclusion, research suggests that in the future, a healthy diet may be considered a reliable tool for maintaining and optimizing our key internal parameters.

## 1. The Biological Cycle of Niacin

Niacin (NA), also known as vitamin B3, nicotinic acid, or vitamin PP, is the most important compound of the B-vitamin complex group. It is an organic and water-soluble vitamin that possesses a dual electric charge. Upon ingestion, niacin is biosynthetically converted to nicotinamide adenine dinucleotide (NAD) [1]. Depending on the exogenous supply, NAD then performs myriad biological functions and has a central role in redox reactions [2].

For example, niacin’s potential to treat pellagra has been recognized since the late 1930s. Pellagra is a remarkable chronic disorder with a clear panel of symptoms [3], representing the final stage of a severe cellular deficiency of niacin. From what is known, substance abuse or digestive disorders [4] that prevent B3 from being metabolized may explain its pathogenesis.

Altschul and his colleagues [5] were the first to evaluate the reliability of nicotinic acid as an anti-dyslipidemic treatment. They demonstrated that niacin (NA) exerts beneficial effects on low-density lipoproteins (LDL), which are rich in cholesterol, by increasing the cholesterol of high-density lipoproteins (HDL).

In the adipose tissue and isolated adipocytes, lipolysis stimulation by catecholamines reduces the production of free fatty acids (FFAs) [6,7]. ^3^H-labeled nicotinic acid is found almost exclusively in adipose tissue, which suggests a modulating role of nicotinic acid in lipolysis [8].

One possible explanation for the antilipolytic effect of nicotinic acid is that it prevents cyclic adenosine monophosphate cAMP from accumulating in adipocytes—more specifically, through the inhibition of adenylyl cyclase. The same research team subsequently made further arguments in relation to the possible underlying mechanism of action, including mention of a receptor coupled to the Gi [9,10,11].

Nicotinic acid stimulates binding of [35S]GTPγS in adipocytes and spleen membranes, but not in other tissues [12]. Following earlier hypotheses suggesting that there were specific binding sites for nicotinic acid on adipocytes and spleen membranes [13], a receptor coupled to the so-called G-protein-coupled receptors was identified in 2003 [14].

The receptor, named hydroxycarboxylic acid receptor 2 (HCA_2_), binds to G-proteins with the expected affinity [14], and this helped to explain how various signals are transduced [15]. Of three homologous receptors [16], HCA_2_ is the only member of the class A rhodopsin/β-adrenergic receptors. Its natural ligand is 3-hydroxybutyrate [17], and its availability to the receptor is mediated by gut microbiota [18].

HCA_2_ is widely distributed within distinct cell types, including the brain and other tissues, where 3-hydroxybutyrate can be used as an alternative energy source [17,19]. Alongside GPR41 and GPR43, HCA_2_ mediates microbe-derived metabolites [20,21,22,23] in starvation. The receptor also regulates neuroimmune mediators of the gut–brain axis (GBA) via GPR109A [24] in neonates and adults that suffer from neurodevelopmental disorders [25,26].

There are three major routes involved in the synthesis of niacin. The salvage pathway produces niacin by converting NAM and nicotinamide ribose (NR) to nicotinamide mononucleotide (NMN) using NAM phosphoribosyltransferase (NamPRTase). NR is phosphorylated by nicotinamide riboside kinase (NRK), while NMN is transformed into NAD under the activity of NMN adenylyltransferase (NMNAT) [27].

While the NRK1 receptor is widely distributed in the body, NRK2 is limited to the muscles, brain, and heart [28]. NMNAT1 and NMNAT2 have identical distributions to NRK1 and NRK2, respectively, although limited to the nucleus. While NMNAT3 is widely expressed in various organs, blood, and skeletal muscle [29], it is located predominantly within the mitochondrial matrix [30].

NA can be synthesized de novo from tryptophan through the kynurenine pathway [31], with 2-amino-3-carboxymuconate-6-semialdehyde (ACMS) being the branching point. ACMS can either be decarboxylated by ACMS decarboxylase (ACMSD) or be subjected to the Preiss Handler pathway, whereby spontaneous cyclization forms quinolinic acid (QA) [32].

NAD is used in the tricarboxylic acid (TCA) cycle as a hydrogen acceptor and mediates the production of NAD(H) from dehydrogenation reactions. Therefore, many of the newly synthesized molecules are redirected and subjected to the oxidative phosphorylation process to produce large quantities of ATP [33].

Mitochondrial localization of NMNAT3 strongly suggests that cellular organelles may be able to utilize NMN from the cytosol when needed. [34]. Besides the nuclear-cytosolic and mitochondrial NAD pools, it has been shown that similar compartments exist at the level of the peroxisomes, endoplasmic reticulum (ER), and Golgi apparatus [35].

NAD is transported from cytosol to peroxisomes through the SLC25A17 transporter, and is then used for the β-oxidation of fatty acids [36]. In the endoplasmic reticulum, NADP is required for the first stage of the pentosophosphate pathway [37], which mediates immunoglobulin-binding protein (BiP) and the translocation of newly synthesized proteins from ribosomes to the endoplasmic reticulum. Their folding in the lumen of the endoplasmic reticulum is then regulated by NAD-dependent mono (ADP-ribozyl) [38]. However, the role of NAD in the Golgi apparatus and the transport mechanism of this remains unknown [39].

There appears to be an increasing volume of literature that explore the close link between exogenous niacin, NAD(P) availability, and dependent enzyme activation [40]. While NAD and its phosphorylated form act as a substrate for oxidoreductase(s) in a catabolic reaction, its precursors are involved in anabolic reactions [2]. The catabolic reactions of NAD give rise to the production of reactive oxygen species (ROS), while NAPD maintains an antioxidant defense.

It has now clear that NAD is also a key element in various intracellular regulation pathways. Three distinct reactions are known in which NAD is cleaved to produce nicotinamide, while the remaining ADP-ribosyl fragment is converted for nicotinamide signaling or attached to proteins. Firstly, NAD glycohydrolases produce ADP-ribose and cyclic ADP-ribose (ADPRc) [33]. These molecules activate calcium channels in the plasma membrane or endoplasmic reticulum, which leads to an increase in the concentration of cytosolic calcium. Secondly, ADP-ribosyl transferases catalyzes changes in the biological activity of both intra- and extracellular proteins. The cellular surfaces of ADP-ribosyl transferases are involved predominantly in immunological functions. Intracellular enzymes have a wide range of functions, including regulation of metabolic enzymes and control of nuclear processes [33].

It can be hypothesized that oxidative stress (OS) is an integrated component, while apoptosis acts as the sentinel. We first review the features of the main NAD-consuming enzymes and their involvement in maintaining homeostasis.

### 1.1. Poly (ADP-Ribose) Polymerases (PARPs)

PARPs are specialized cell signaling enzymes used to catalyze the transfer of a specific ester to targeted DNA proteins [41]. ADP-ribosylation (ADPr) is a reversible post-translational modification (PTM) used to conserve ADP-ribose in pro- and eukaryotes [42,43,44]. It is produced as a result of ADP-ribosyltransferase (ART) activity and defined by a transfer of the ADPr from NAD to specific substrates such as N-, O-, and S- [45].

Sixteen members of the PARP family have been identified to date, and are defined by distinct groups of genes with a homologous catalytic domain (CAT). PARP-1 is predominantly involved in the DNA repair process, cell proliferation, and apoptosis, as are PARP-2 and PARP-3 but to a lesser degree, respectively [41,46].

It should also be noted that PARP-1–5 possess glutamate (Glu988); whereas PARP-6–16 are generally considered to be mono (ADP-ribose) polymerases, with the exceptions of PARP-9 and -13, which are inactive [47]. Their involvement in the maintenance of DNA integrity is presented in Table 1.

### 1.2. Sirtuins

Analogous to ADPr, protein acetylation is a PTM that modulates key protein functions including their interaction and stability, but also their role in DNA recognition and catalytic activities [49]. Acetylation and deacetylation of N-epsilon lysine residues are catalyzed by histone acetyltransferases (HATs) and histone deacetylases (HDACs) [50].

There are four classes of HDACs based on phylogenetic analysis of all HDAC-related proteins [51] and including sirtuins, which belong to class III. There are seven sirtuins, which are distributed across three intracellular compartments. SIRT1 and SIRT2 can be found in the nucleus and cytoplasm; SIRT6 and SIRT 7 are found in the nucleus alone, while SIRT3, SIRT4, and SIRT5 are found in the mitochondria [52].

The only difference between SIRT and the other HDACs is their ability to catalyze the deacetylation of proteins using NAD and to play a definitory role in various crucial processes (Table 2).

### 1.3. Cluster of Differentiation: 38 and 157

CD38 and CD157 are usually involved in calcium signaling and the cell cycle through production of the second cADPR messenger [55] and ADP-ribosyl cyclase-produced glycoproteins. Approximately 100 molecules of NAD are required to produce 1 molecule of cyclic ADP ribose (cADPR) [56].

Quarona et al. [57] detailed the main features of both CD38 and CD157 in their review, focusing specifically on coding genes, distribution within cells, and the interplay between innate and adaptive immunity.

Ca^2+^ signaling is one of the more important signal transduction mechanisms, strictly controlled by mechanisms which allow mobilization of the calcium from Ca^2+^ deposits [58]. Cyclic ADPR is derived directly from NAD, while NAADP is a derivative of NADP [59]. Both molecules differ in their interaction with the calcium channels [60].

cADPR triggers Ca^2+^ release using Ca^2+^ (CICR) and its interactions with the receptor for ryanodine (Ryr) [61,62]. Moreover, CD38 can produce ADPR from NAD, which binds to the plasma membrane of TRPM2 and regulates the influx of extracellular Ca^2+^ [63]. Therefore, it is not surprising that poor Ca^2+^ signaling has been associated with tumorigenesis [64].

## 2. Involvement of NAD and NAD(P) in Apoptosis and Gene Expression

When compared to the literature available on the role of PARP-1 as a mediator of necrosis [65,66], there is only a limited number of studies highlighting the impact of NAD in apoptosis.

Inhibition of the kynurenine pathway [67] is characterized by severe apoptosis, whilst depletion of NAD [68,69] is characterized by reduced apoptosis. This is the result of abnormal expression of p53 [70] and the autophagy process [71]. Interestingly, OS has been shown to inhibit the entire apoptosis process [72,73], while exogenous supply with NMN can modulate cell death [74] and cause the early depletion of NAD/NADPH reserves [75].

NAD can modulate apoptosis through several mechanisms because (1) it mediates cellular energy metabolism and influences the onset of different types of cell death; (2) the NADH/NAD ratio constitutes a major index of cell reduction power, affecting mitochondrial transition permeability [76]; (3) the NAD level modulates the activity of caspase-dependent endonuclease DFF40 [77]; (4) NAD-dependent sirtuins mediate apoptosis [78].

However, some studies have revealed a protective role, such as with mitochondrial NADP isocitrate-dependent dehydrogenase (IDH2), which acts against cellular apoptosis induced by various insults—for example, apoptosis induced by cadmium exposure [79]. Administration of a competitive inhibitor of IDH2 (oxalomalate) leads to exacerbated apoptosis induced by ionizing radiation in murine models [80], and also to modulation of the activity of IDH2 in HEK293 cells, which was significantly impairing in the case of high-glucose-induced apoptosis [81].

Many studies have demonstrated the key role of NADPH oxidase in cell death, in both in vitro and in vivo conditions [82]. For example, NADPH oxidase activity in astrocytes mediates neuronal death induced by β-amyloid plaques [83]. NADPH oxidase also plays a crucial role in the generation of reactive oxygen species (ROS) in neurons when deprived of oxygen and glucose in an in vitro model of cerebral ischemia [84]. Due to the critical role of OS in cell death [85], further research is required to strengthen our understanding of the role played by NADPH in cell death.

The mechanisms mentioned previosuly can be explained by the fact that PARP-1 plays an important role in mediating gene expression through a number of mechanisms:

(1) PARP-1 can affect multiple transcription factors, including activator protein 1 and 2 (AP-1/2), kappa B nuclear factor (NFkB), tumor protein p53, protein 1 related to the sensitive element of cAMP, the sex-determining region of the Y chromosome (Sry), and hypoxia inducing factor 1 (HIF1) [86,87,88,89,90,91];

(2) The binding of PARP-1 to nucleosomes can reversibly modulate the structure of chromatin in a NAD-dependent manner: its binding can promote the formation of structured chromatin, which is repressed after transcription, while an auto-poly (ADP-ribosyl)ation area in the presence of NAD produces PARP-1 dissociation from chromatin and leads to the formation of a decoded chromatin structure which is active for transcription [88,92];

(3) PARP-1 produces the poly (ADPribozyl)ation of the histone H1, causing chromatin decondensation [93,94];

(4) PARP-1 can dependently inhibit RNA polymerase II transcription (Pol II) [95,96,97];

(5) PARP-1 may directly affect gene expression by binding to the promoters of certain genes, such as isoform nitric oxide synthase (iNOS) and chemokine (reason C-X-C) ligand 1 [98,99];

(6) PARP-1 can suppress gene expression by modulating DNA methylation [100,101,102,103];

(7) The consumption of PARP-1-dependent NAD can inhibit gene expression by influencing NAD-dependent sirtuins that can modulate the activity of transcription factors [53].

Studies suggest that sirtuins may also mediate gene expression through multiple pathways:

(1) Silent information regulator 2 (SIR2) from yeasts and SIRT1 in mammals can cause histone hypoacetylation and abnormal gene expression by promoting heterochromatin assembly [53];

(2) SIRT1 causes acetylation of certain factors involved in transcription, such as tumor protein p53 [104], the forkhead proteins [105,106], nuclear factor kappa-light-chain-enhancer of activated B cells (NF-κB) [78], tumoral protein p73 [107], and trans-activator of transcription (tat) [108];

(3) SIRT7 is an activator of RNA polymerase I (Pol I)-mediated transcription [109];

(4) SIRT1 inhibits Pol I-mediated transcription through the deacylation of TAFI68 [110].

## 3. Mechanism of NAD in the DNA Damage Response

Age has been identified as one of the main factors contributing to an increase in insults and the accumulation of damage to DNA. Furthermore, there a correlation has been found between age, depletion of NAD (reduction of NAMPT activity), and increased levels of NAD(H) [111].

The againg process is almost always associated with a variety of diseases [112], and recent studies have highlighted the importance of NAD synthesis in the pathophysiology of the certain age-related disorders [113]. In vivo imaging data have revealed that NAD levels and mitochondrial function differ in the elderly when compared to the young [114].

It was found that expression of NAMPT decreases with age and may explain why the inhibition or overexpression of NAMPT can accelerate or decelerate age-related changes [115,116,117]. Moreover, there is a correlation between NAMPT expression and circadian oscillation. Since oscillation reduces with age, it is theorised that the total level of NAMPT can also be influenced [118].

As the accumulated DNA damage increases in the elderly, PARP-1 activation causes a decrease in NAD levels and simultaneously alters metabolism. However, PARP-1 helps to maintain genome integrity, and its activation during the aging process seems to be multifaceted [119].

In the case of murine strains, aging is associated with an increase in CD38 activity, which negatively correlates with levels of NAD and mitochondrial activity. This response can be at least partially attributed to a decrease in SIRT3 activity. In contrast, CD38-knockout murine models appear to be protected against age-related decline in NAD and mitochondrial activity [120]. As the activity of sirtuins decreases continuously, the reduction of NAD levels constitutes the main cause of the deficiency of sirtuin activity and possibly the development of age-related diseases [121].

All the mechanisms mentioned above promote the production of reactive oxygen (ROS) and nitrogen species (RNS). Although RNS and ROS can directly affect DNA, cells are equipped with complex mechanisms to ensure some protection of genome integrity. While base-excision repair (BER), nucleotide-excision repair (NER), and mismatch repair (MMR) help to repair single-stranded breaks (SSBs), homologous recombination (HR) and non-homologous end joining (NHEJ) are the guardians against double-stranded breaks (DSBs) (Table 3).

As a result, one can conclude that DNA is very susceptible to insult, and so the following section focuses on how components such as oxidative stress can further disturb human gastrointestinal microflora and IVF outcomes.

## 4. Does Oxidative Stress Influence Fertilization Rate?

**Figure 1:**
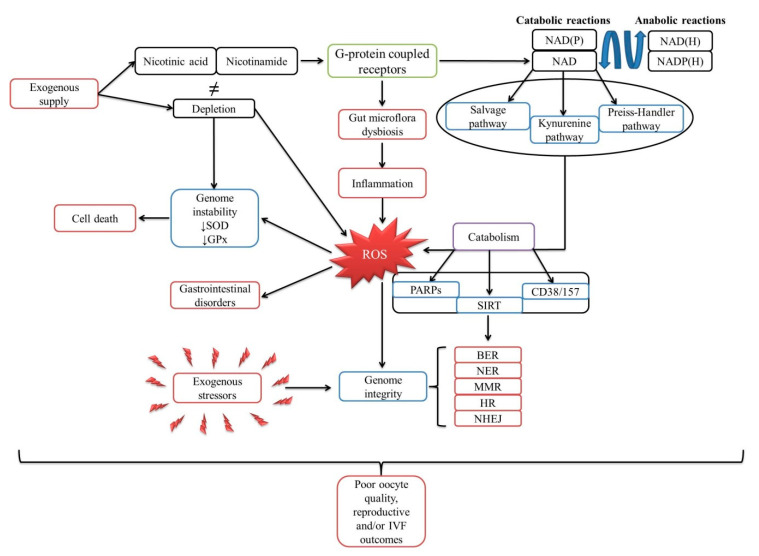
Schematic representation of niacin’s biological cycle. Once ingested, the G-protein-coupled receptors initiate intracellular responses, and niacin is converted into four specific precursors involved in both anabolic and catabolic reactions. However, if the gut microflora is disrupted, it produces a large amount of reactive oxygen species, which influences genome structure. If a depletion occurs, NAD is synthesized through three specific pathways, which strongly correlate with three NAD-consuming enzymes, but also with the production of reactive oxygen species. Subsequently, five highly specialized mechanisms ensure genome integrity against any exogenous or endogenous stressor(s). In the absence of an exogenous supply of niacin, there is a gradual, progressive instability of the genome, characterized by the inability of the antioxidant system to act efficiently, which ultimately leads to cell death. Cumulatively, all of these aspects could ultimately affect the viability of in vitro fertilization protocols through a deterioration of oocyte quality.

Relatively few studies have been conducted over the years to look directly at the impact of ROS on oocyte quality, and therefore limited data are available. A schematic representation of possible mechanisms, summarizing all those described in previous sections, is presented in Figure 1.

Tulić et al. [123] demonstrated that ROS do not negatively influence IVF outcomes, but significant differences have been noted between protocols. A GnRH-agonist protocol has been proven to be more reliable in terms of developing mature oocytes and fertilization when compared with a GnRH antagonist. There were no differences between the number of biochemical pregnancies, miscarriage, or live birth rate. By measuring superoxide dismutase (SOD), malondialdehyde (MDA) and SH in serum, the authors concluded that the SOD was significantly lower in contrast with MDA and SH.

On the other hand, Siristatidis et al. [124] reported opposing findings and showed that following measurement of ROS in blood samples at oocyte retrieval and in follicular fluid (FF), there was no association between ROS and the quality of embryos following IVF.

However, evaluating antioxidant status may help to predict IVF outcome. This was the case reported by Nishihara et al. [125] in a study where they have showed that patients with a low fertilization rate had also low levels of glutathione (GSH) following intracytoplasmic sperm injection (ICSI). In turn, those with a high fertilization rate had high levels of 8-Oxo-2′-deoxyguanosine (8-OHdG) in the FF. The authors also suggested that OS in infertile women are associated with endometriosis.

Their observations were reinforced by the results of Borowiecka et al. [126]. They revealed that an elevated FF lipid level and the process of protein peroxidation could negatively influence IVF outcome after analysis of thiobarbituric-acid-reactive substances (TBARS) in pregnant women.

In addition to disturbing the body’s internal parameters, heavy [127] and trace elements [128] act as exogenous stressors, as they cause an increase in the production of specific oxidative biomarkers. Possible therapies include treatment with micronutrients that may optimize the host microenvironment against reactive oxygen species [129].

It has been demonstrated that niacin supplementation could have an important role in treating premature ovarian failure (POF). Researchers have also found that B3 promotes follicular growth and an increase in the level of two markers, both involved in mediating apoptosis in cultured cell lines from mice [130].

## 5. Is There a Relationship between Oxidative Stress and Gut Dysbacteriosis?

Considering the myriad functions fulfilled by the gut microflora [131] and the close link between the brain [25,132] and digestive tract [33,133], there have been relatively few reports aiming to demonstrate how OS gradually induces dysbiosis. This section focuses on the studies and hypotheseses that clearly demonstrate how OS disrupts host eubiosis [134].

*Lactobacillus plantarum* YW11 [135], *Lactobacillus plantarum* CCFM10 [136], and *Enterococcus durans* MTCC 3031 [137] were found to restore microbial integrity in aging murine models through normalization of the redox ratio. On the other hand, gut metabolism in mice could be disrupted following administration of antibiotics, which was characterized by a shift of the redox potential after just 24 h [138].

Diet also has a pivotal role in optimizing the function of gastrointestinal microbiota. The rodent diet consists of oxidized animal proteins and results in significant impairment of the mucosal barrier. The disbalance is characterized by a reduction of several beneficial strains such as *Akkermansia*, *Lactobacillus*, and *Desulfovibrio*, to the detriment of proinflammatory strains like *Escherichia*–*Shigella* and *Mucispirillum* [139].

Extruded sorghum flour (ESF) has been found to improve the intestinal microbiota of obese rats by enhancing the proportion of the Bacteroidetes phylum, and lowering that of the Firmicutes phylum. ESF also diminished the concentration of a series of proinflammatory biomarkers, and subsequently increased the overall antioxidant capacity [140].

A similar pattern was also found in human patients with cardiovascular or kidney disease. Measurements of plasma and serum biomarkers have indicated fluctuation between strains, low richness, and increased levels of OS, proinflammatory cascades, and endotoxemia [141,142].

On the other hand, silver nanoparticle (AgNP) usage as a potential treatment for colorectal cancer (CRC) negatively influenced the metabolism of *Enterococcus durans*. Even in the presence of low AgNP concentrations, increased intracellular hydroxyl radical and extracellular folic acid concentrations were observed [143]. Environmental arsenic exercises a similar effect upon gene(s) expression, modifying the overall microbial diversity and the pathways of synthesis involved in a variety of functions in mice [144].

It is certain that in such stressful conditions, the imbalance created between pro- and antioxidants is significant, with OS playing the role of the main pathological substrate for a number of neurological disorders [145,146,147,148,149,150,151].

Unfortunately, the number of studies aiming to demonstrate how OS perturb these microbial associations are limited. Based on these considerations, we attempt below to explain the loss of host eubiosis through the prism of the “global pandemic” caused by the novel coronavirus.

### The Possible Interconnections between Gastrointestinal Deficiencies and the Novel Coronavirus

Continuing with this concept, we aim to highlight the crucial aspects regarding the involvement of the novel coronavirus in the disturbance of host eubiosis, resulting in a proinflammatory cascade.

A recent review discussed about the crosstalk between lung and gut microbiota in the elderly. According to the authors, age could be responsible for the high predisposition of elder people to SARS-CoV-2 infections. It seems that age is directly correlated with an increased gut permeability and the associated gastrointestinal deficiencies, which suggests a potential for fecal–oral transmission [152].

Even though no study has been conducted on this topic, early studies on COVID-10 highlighted a low [153,154,155,156] to medium [157,158,159,160] incidence of gastrointestinal deficiencies (Table 4). The most common symptom was diarrhea [161,162,163,164], which suggests a potential route of action of COVID-19 at the level of digestive tract.

Based on these initial considerations, it should be also mentioned that gastrointestinal (GI) dysfunctions (e.g., diarrhea) are part of a cluster of specific symptoms displayed by patients suffering from irritable bowel syndrome (IBS) [165]. We have summarized (Table 4) data related to these abnormalities that may result from disturbances along the gut–brain axis (GBA) [166].

**Table 4:** Frequency of the most uncommon gastrointestinal symptoms displayed by SARS-CoV-2-infected patients.

Number of patients included	**Number of Patients Included**	**Occurrence**	**Reference**
	99	2 (2%)	[155]
	41	1/38 (3%)	[154]
	1099	42 (3.8%)	[153]
	62	3 (8%)	[156]
	651	74 (8.14%)	[157]
	191	9 (5%)	[158]
	138	14 (10.1%)	[159]
	95	58 (24.2%)	[160]
	274	77 (28%)	[167]

Over the years, analytical biochemical analysis has begun to be used as an integrated component alongside molecular biology. Thus, the optimization of techniques (e.g., ELISA) has allowed fecal calprotectin (FC) to evolve as a reliable biomarker that allows the identification of intestinal inflammation [168].

Using a cohort consisting of 40 patients, Effenberger et al. [169] aimed to measure the level of FC. According to their data, 45% of patients did not report GI symptoms, in 55% of cases diarrhea has ceased after two days, and in nine patients, diarrhea persisted beyond the two days. They demonstrated that patients with or without diarrhea had FC levels significantly correlated with interleukin-6 (IL-6) concentration, but not with that of C-reactive protein (CRP) or ferritin.

Contrary to the hypothesis that people with additional diseases are more prone to infection, those with IBD were not at increased risk of COVID-19 and the associated mortality in one observational, case-series study [170]. What is certain is that there is controversy around this topic, because we also identified antithetical studies.

For example, we encountered a report of a patient diagnosed with an acute, severe ulcerative colitis (UC) flare who died. As part of the management of the IBD, the patient received high intravenous dose of corticosteroids. He subsequently developed pneumonia and was found to have COVID-19 based on a nasopharyngeal swab [171].

To date, only two large clinical trials have been conducted with the aim of assessing GI symptoms and detecting the virus following the analysis of fecal samples [157,160]. Among all patients (n = 651), Jin et al. [157] concluded that on the basis of GI dysfunctions, only 11.4% (74 cases) had digestive problems, of which 28% did not manifest any respiratory difficulties. Lin et al. [160] offered a more conclusive perspective, showing that 61.1% (58/95) of patients had GI dysfunctions, the most frequent being diarrhea, nausea, vomiting, and liver impairment. Thus, it was possible to identify the niches in which SARS-CoV-2 was detected [160], with it being observed even in the stool samples on Day 7, up to twelve days [156,172,173,174].

It has been hypothesized that SARS-CoV-2 could even be transmitted postmortem [175], persisting in the body after clearance of the respiratory tract [176], with viral signatures being found 1 month later after admission [174].

There is an increasing trend in the literature regarding the underlying mechanism or possible interconnections between COVID-19 and the myriad microscopic entities that are gathered at the level of every human gastrointestinal tract [177].

In that context, an immunopathological mechanism and novel therapeutic targets have been revealed. Using a machine-learning model dedicated to exploring core microflora in order to predict the outcome, it was possible to identify an increased expression and exacerbated levels in fourteen proinflammatory cytokines [178,179].

Mechanically speaking, the main gateway of the virus could be represented by the angiotensin system, or, more precisely, the angiotensin-converting enzyme 2 (ACE2) as a viable receptor [180]. ACE2 can mediate intestinal inflammation [181], which explains its high expression on the epithelial cells [182] and lymphocytes’ exhaustion status [183,184]. By analyzing single-cell RNA sequencing data, it was discovered that ACE2 is highly expressed in the small intestine [164].

The latest reports have revealed the importance it has enteric microflora in regulating the neuroimmune network [185,186,187]. SARS-CoV-2 is not only dependent on the presence of the ACE2 receptor. The structural composition and proportions by which ACE2 is expressed are distinct in the digestive tract (e.g., esophagus, gastric, ileum, and colon). Therefore, this could explain how the S protein is cleaved on the cell membrane and the interplay between transmembrane protease, serine 2 and 4 (TMPRSS2/4) [188] and how the gut enterocytes are gradually infected [189].

Even though PCR-based methods have provenr efficiency and are used at a global scale, a recent report demonstrated that eight out of ten “infected” children could be false positives. Even when initial nasopharyngeal testing was negative, rectal swabs have proven the opposite [190]. Through electron microscopy, it was possible to detect patients that did not have diarrhea, but were positive, which strongly suggests once again an oral–fecal transmission [191].

At approximately three weeks after onset, in all patients (n = 285) in one study, it was reported that a seroconversion of immunoglobulin G (IgG) and immunoglobulin M (IgM) took place [192].

Because there is no active treatment against this infection, it has been proposed that mouth rinses containing β-cyclodextrin combined with citrox could exert a beneficial effect, but this remains in a theoretical stage [193], as does the usage of hydroxychloroquine (HCQ) [194].

On the other hand, mouthwash containing chlorhexidine was associated with a richness of several strains of Firmicutes and Proteobacteria, but negatively correlated with Bacteroidetes, Saccharibacteria, Candidate division TM 7, Candidate division SR1, and Fusobacteria, inducing massive shifts among the microorganisms found inside the oral cavity by leading to a more acidic pH [195].

Currently, there have been no studies aiming to demonstrate the participation of COVID-19 in the production of ROS. However, dysbacteriosis is characterized by a proinflammatory cascade, which has as a consequence the persistent production of ROS. Thus, it can be hypothesized that COVID-19 may cause an increase in the production of ROS through dysbacteriosis as a consequence of the proinflammatory state. ROS production can be mediated through adequate nutrition, or, more precisely, through probiotics that strengthen the intestinal epithelium and thus prevent any possible adhesion of pathogenic entities [196].

## 6. Conclusions

It can be concluded based on the aspects detailed in this minireview that niacin indeed plays a pivotal role in homeostasis. It is an essential vitamin for the maintenance of an optimal internal environment, and for the functionality of the main NAD-consuming enzymes and mechanisms. Niacin and its derived forms are involved in a multitude of biological functions such as gene(s) expression and the apoptosis process. It seems that gut microflora modulates the availability of niacin once ingested, with gut metabolism proving to be directly correlated with a fulminant production of reactive oxygen species in case of a dysbiosis. Studies performed on COVID-19 have also offered novel insights regarding the underlying interaction mechanisms between COVID-19, the gut, and proinflammatory cascade(s). However, unanswered questions remain, as do new challenges to come. Regardless of the current diagnostic approaches, we have also presented cases where patients were initially false negative, which is not uncommon. Thus, paying more attention to analysis performed on stool samples or swabs may present a novel diagnostic tool in the identification of COVID-19, with the aim of improving the overall sensitivity of screening measures. The importance of homeostasis can also be seen with in vitro fertilization protocols, where a possible loss of homeostasis influences the fertilization outcome, and, even here, there are controversies regarding the antagonistic involvement of oxidative stress in such protocols. It follows that a correlation between oxidative stress and oocyte quality and overall fertilization rate exists, which is why additional studies are needed. It can be concluded that an exogenous supply of niacin has proven to be crucial, and not only for maintaining genome integrity against insults inflicted by OS, because of its branched implications in gene(s) expression, apoptosis process, host eubiosis, and IVF outcome. On the other hand, OS production may play an important role against infection, such as that caused by the novel coronavirus, but this topic is controversial and reports do not yet exist in the current literature.

## Figures and Tables

**Table 1:** Main biological functions fulfilled by the PARP-family enzymes.

PARP	Subcellular Localization	Biological Role(s) [48]
PARP-1	Nucleus	Gene regulation DNA damage response
PARP-2	Nucleus	DNA repair Base-excision repair
PARP-3	Nucleus	DNA repair
PARP-4	Cytosol	DNA repair Cell death Inflammation
PARP-5a	Nucleus, cytosol	Wnt signaling pathways Cell division mRNA and protein transport Telomerase regulation Protein ubiquitination
PARP-5b	Nucleus, cytosol	Wnt signaling pathways Telomerase regulation Protein ubiquitination
PARP-6	unknown	ADP-ribosyltransferase activity
PARP-7	unknown	Hormonal processes Embryonic development and morphogenesis
PARP-8	unknown	ADP-ribosyltransferase activity
PARP-9	Nucleus, cytosol	DNA repair Cell migration Response to interferon γ
PARP-10	Nucleus; cytosol	Cell proliferation Chromatin assembly regulation
PARP-11	unknown	ADP-ribosyltransferase activity
PARP-12	Nucleus	ADP-ribosyltransferase activity Nucleic acid binding Zinc ion binding
PARP-13	Cytosol, nucleus, plasma membrane, Golgi apparatus	Innate immune response
PARP-14	Cytosol, nucleus, plasma membrane	Transcriptional regulation
PARP-15	Nucleus	Transcriptional regulation
PARP-16	Endoplasmic reticulum Nuclear membrane	Response to unfolded proteins

**Table 2:** Biological functions fulfilled by sirtuins in mammals.

Sirtuin	Biological Role	Reference
SIRT1	Metabolism Stress	[53,54]
SIRT2	Cell cycle	
SIRT3	Thermogenesis ATP production	
SIRT4	Insulin secretion	
SIRT5	Urea cycle	
SIRT6	Base-excision repair and metabolism	
SIRT7	rDNA	

**Table 3:** The main biological mechanisms involved in DNA repair processes.

DNA Repair Mechanism	Involvement	Reference
Base-excision repair	DNA polymerase β interaction	[122]
	X-ray repair cross-complementing protein 1 recruitment	
	Activator protein 1	
	PARP-1 binding (PARP-2)	
	X-repair cros-complementing protein 1 interaction (PARG)	
	DNA ligase III binding (PARP-1)	
Nucleotide-excision repair	DNA repair protein comlementing XP-A cells association (PARP-1)	
	DNA damage-binding protein 2 (PARP-1)	
	Xeroderma pigmentosum, complementation group C (PARP-1)	
Mismatch repair	DNA mismatch repair protein MutS Homolog 3 interaction (PARP-1)	
Homologous recombination repair	Breast cancer type 1 susceptibility protein recruitment (PARP-1)	[33]
Non-homologous end-joining repair	DNA-dependent protein kinase, catalytic subunit, Ku80/Ku70 heterodimer interaction (PARP-1)	
